# Supplementary material for: Study of UHMWPE Fiber Surface Modification and the Properties of UHMWPE/Epoxy Composite
Source: Polymers (Basel). 2020 Mar 1;12(3):521. doi: 10.3390/polym12030521 (PMC7182862; doi:10.3390/polym12030521)
Supplement: Supplementary file 1 [file polymers-12-00521-s001.pdf]

polymers

Supplimentary File

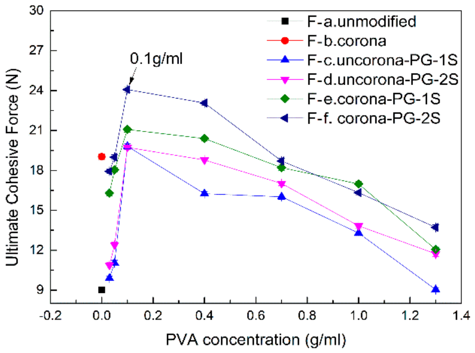

Figure 1. Ultimate Cohesive Force of the composite with different PVA concentration of the six methods.

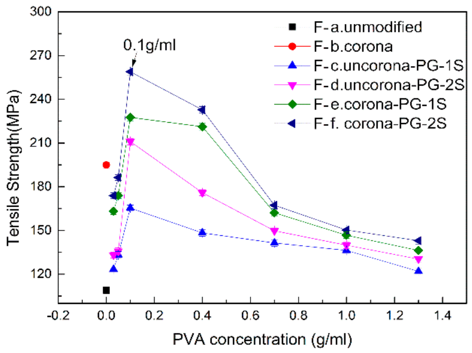

Figure 2. Tensile Strength of the composite with different PVA concentration of the six methods.

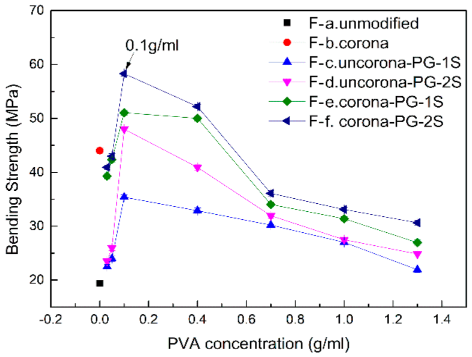

Figure 3. Bending strength of the composite with different PVA concentration of the six methods.

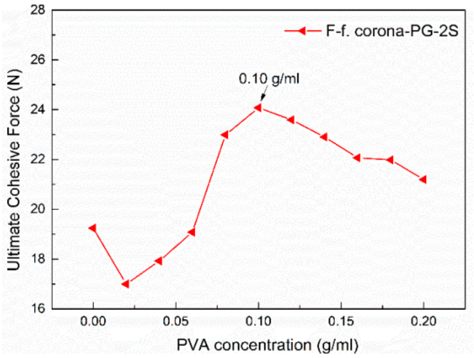

Figure 4. Ultimate Cohesive Force the composite treated by Corona-PG-2S with smaller PVA concentration gradient.

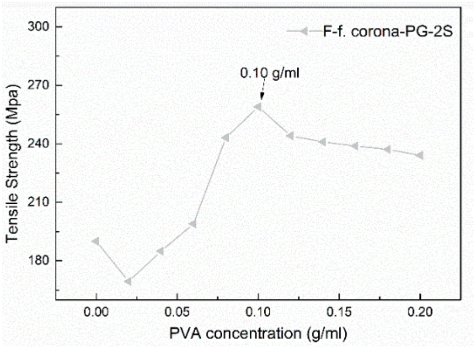

Figure 5. Tensile Strength the composite treated by Corona-PG-2S with smaller PVA concentration gradient.

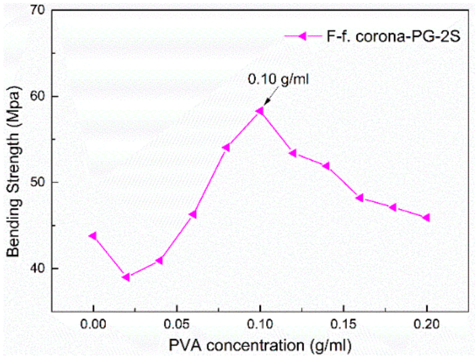

Figure 6. Bending strength the composite treated by Corona-PG-2S with smaller PVA concentration gradient.
